# A Multitude of Neural Representations Behind Multisensory “Social Norm” Processing

**DOI:** 10.3389/fnhum.2018.00153

**Published:** 2018-04-23

**Authors:** Felipe Pegado, Michelle H. A. Hendriks, Steffie Amelynck, Nicky Daniels, Jessica Bulthé, Haemy Lee Masson, Bart Boets, Hans Op de Beeck

**Affiliations:** ^1^Department of Brain and Cognition, KU Leuven, Leuven, Belgium; ^2^Center for Developmental Psychiatry, Department of Neurosciences, KU Leuven, Leuven, Belgium; ^3^Leuven Autism Research Consortium, KU Leuven, Leuven, Belgium

**Keywords:** multisensory, audio-visual, multivoxel pattern analysis, orthogonal design, hierarchical brain, social norm, mentalizing

## Abstract

Humans show a unique capacity to process complex information from multiple sources. Social perception in natural environment provides a good example of such capacity as it typically requires the integration of information from different sensory systems, and also from different levels of sensory processing. Here, instead of studying one isolate system and level of representation, we focused upon a neuroimaging paradigm which allows to capture multiple brain representations simultaneously, i.e., low and high-level processing in two different sensory systems, as well as abstract cognitive processing of congruency. Subjects performed social decisions based on the congruency between auditory and visual processing. Using multivoxel pattern analysis (MVPA) of functional magnetic resonance imaging (fMRI) data, we probed a wide variety of representations. Our results confirmed the expected representations at each level and system according to the literature. Further, beyond the hierarchical organization of the visual, auditory and higher order neural systems, we provide a more nuanced picture of the brain functional architecture. Indeed, brain regions of the same neural system show similarity in their representations, but they also share information with regions from other systems. Further, the strength of neural information varied considerably across domains in a way that was not obviously related to task relevance. For instance, selectivity for task-irrelevant animacy of visual input was very strong. The present approach represents a new way to explore the richness of co-activated brain representations underlying the natural complexity in human cognition.

## Introduction

Humans have an extraordinary capacity to integrate complex multidimensional information. A first challenge arises from the brain’s hierarchical structure, with multiple dimensions being represented at different levels (Op de Beeck et al., 2008). For example, to process the visual dimensions aspect ratio and symmetry, a nonlinear combination of simple features computed in earlier sensory levels is needed. Humans excel in such nonlinear tasks, while animals like rats fail (Bossens and Op de Beeck, 2016).

A second challenge involves the combination of information across different sensory systems (McGurk and MacDonald, 1976; Pourtois et al., 2005). For instance, to read printed words, the human brain associates visual information of letters to auditory representations of sounds and higher-order representations of meaning (Dehaene et al., 2010). A third challenge involves the combination of information from multiple sensory systems as input for more high-level cognitive processing. For instance, the understanding of everyday social situations, a kind of “social reading”, requires the simultaneous processing of visual cues—e.g., face expressions (Vuilleumier and Pourtois, 2007), auditory cues—e.g., prosody (Bestelmeyer et al., 2014; Sammler et al., 2015) and higher-order social information—e.g., mentalizing others’ goals, desires or mental states (Frith and Frith, 2006; Mitchell, 2009; Schurz et al., 2014). In the scientific literature all these different processes are typically studied in separation.

In the current study, we aimed to uncover the multiple representations that are activated in complex social tasks. We focus upon a neuroimaging paradigm which implements a complex example of social understanding, requesting people to infer how most people would judge the congruency of vocal reactions to visual scenes, a high-level social norm inference task (see Pegado et al., 2017). Here we analyze the data in such a way that we can disentangle multiple representations in different systems and hierarchical levels, at the same time. In other words, instead of focusing upon either visual, auditory, or social processes separately, we aim to capture them all at once. Our goal is to provide a demonstration of principle that it is possible to investigate multiple neural systems at different hierarchical levels simultaneously in an ecologically valid way. Accordingly, we expect to find a variety of neural representations in a network of brain regions. In visual regions, we expect a hierarchy of representations, from low-level visual characteristics in primary visual cortex (Kamitani and Tong, 2005; Kay et al., 2008) to high-level category-related properties in lateral and ventral occipito-temporal cortex (Kriegeskorte et al., 2008b; Bracci and Op de Beeck, 2016). In auditory regions, we expect to find representations of multiple aspects of auditory stimuli, including low-level acoustic properties and higher-level emotional content (Formisano et al., 2008; Ethofer et al., 2009). Further, high-level inferences of social norm are expected to be represented in the core mentalizing network, including the temporo-parietal junction (TPJ), precuneus (PC) and medial prefrontal cortex (mPFC; Amodio and Frith, 2006; Frith and Frith, 2006; Mitchell, 2009; Schurz et al., 2014). In an earlier manuscript we already reported the representation of social congruency in these data. Here we focus on the many other representations that are co-activated and the resulting similarities between brain regions in which information they represent and which not.

## Materials and Methods

### Participants

Twenty-five healthy subjects (seven females, 23.16 ± 3.32 years old, seven left-handed) took part in this functional magnetic resonance imaging (fMRI) study. They all reported normal or corrected-to-normal vision, normal hearing and no neurological or psychiatric disorders. They received a financial compensation for their participation. The study was approved by the Medical Ethics Committee UZ/KU Leuven University and all methods were performed in accordance with the relevant guidelines and regulations. Prior to scanning, all participants provided written informed consent in accordance with the Declaration of Helsinki.

### Task

Participants were lying in the scanner while watching a visual display and hearing auditory input through headphones. They were instructed to imagine they were seeing images (photographs) together with other unknown people. For each image, after a short delay, participants hear a vocal reaction (emotional utterance) that could be more or less congruent with the particular scene. The task was to evaluate the congruency of the vocal reaction in relation to the visual context. Yet, they did not have to judge this congruency from their own personal perspective, but instead they were explicitly instructed to evaluate whether most people would consider this vocal response appropriate or not (mentalizing the “social norm”) and respond accordingly. The two assigned buttons (congruent vs. incongruent) were switched after three runs out of six, and the assigned order was balanced across subjects. Note that our paradigm was previously validated at the behavioral level (Pegado et al., 2017). Indeed, as there is no “correct answer” for this task but only subjective judgments, we analyzed the subjects’ agreement on these subjective judgments of audio-visual congruency. Crucially, we showed that when subjects performed the task inferring what most people would answer (instead of themselves) the correlation of the response choices across subjects increased, relative to when participants judged using their own perspectives. This demonstrates the sensitivity of the paradigm for the perspective taken by the participants. Here we used the “social norm perspective” task, as this particular aspect of mentalizing (infer the “common knowledge”) has not been investigated previously. We used a multisensory social situation to increase ecological validity (as our everyday life environment is multisensorial) and to able to capture multiple neural systems representations at once. In this way, we could obtain a richer representation of the brain activity during this complex cognition.”

### Visual Stimuli

**Figure 1:**
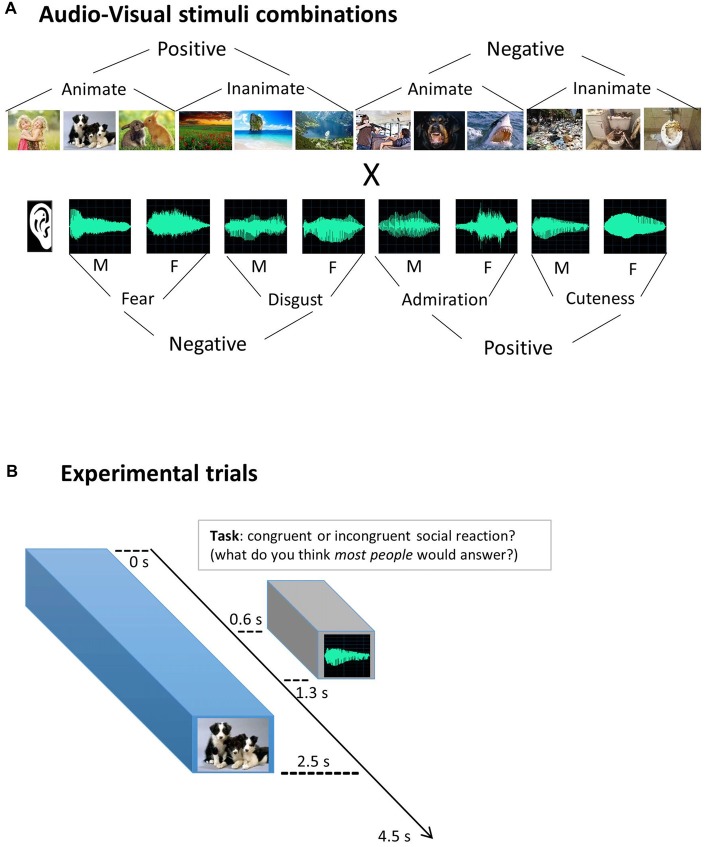
Experimental design. **(A)** Audio-Visual stimuli combinations. Visual images were combined with auditory stimuli (vocal utterances), simulating social reactions for different scenarios. In every run, all 12 images were combined with all eight vocal utterances, enabling an orthogonal analysis of each dimension: visual, auditory and social congruency. F = female voice; M = male voice. **(B)** Experimental trials. Participants were asked to judge the appropriateness of the social reactions, responding “congruent” or “incongruent” with assigned buttons. They were explicitly instructed not to use their own personal opinion (self-reference) but to infer what most of people would think about the social appropriateness (“social norm” mentalizing) and to respond accordingly. To mimic a natural timing of social reactions, vocal utterances started 0.6 safter the visual scene. Respecting the International Affective Picture System (IAPS) policy, the images presented here are not the original ones.

Twelve images were selected from the standardized and widely used emotional pictures set International Affective Picture System (IAPS; Lang et al., 2008), based on extreme valence ratings (positive vs. negative) and on animacy categorization (animate vs. inanimate). The six animate (e.g., humans, animals…) and the six non-animate pictures (e.g., landscapes, objects…) were orthogonal to the image valence, with half of them rated positively (e.g., happy baby), and half of them negatively (e.g., people being threatened with a gun). Based upon the quality of the brain responses evoked by a larger dataset of 24 IAPS images present in the pilot fMRI study (see infra), this final set of 12 images was selected: numbered 2341, 1710, 1750, 5760, 5825, 7492, 3530, 1300, 1930, 9290, 9300, 9301 in the IAPS database. Figure 1 provides an illustration of the stimulus set. As IAPS policy requests not to publish the original images, we depicted here a series of similar image examples found on the internet, in the same order as the original ones mentioned above.

### Auditory Stimuli

Eight different non-verbal vocal utterances were used, inspired in previous work (Sauter et al., 2010). They express four different emotional reactions that could be more or less congruent with the pictures previously selected. Utterances expressing disgust, fear, admiration and cuteness, were recorded in an expressive but still natural manner (not an exaggerated caricature). Each emotional vocalization was performed by one male and one female actor. They were recorded in a sound-proof room at 96 kHz sampling rate and 32-bit resolution and were down-sampled to 44 kHz and 16-bit mono-recordings to reduce the size of the audio files. All stimuli had a fixed duration of 700 ms and an equivalent total Root Mean Square (RMS) power (–17.4 ± 0.17 dB). Stimuli were slightly manipulated in Cool Edit Pro software and Adobe Audition CC 2015 software. Identical 600 ms silent periods were added before the onset of each auditory stimulus to create a natural delay from the visual stimulus onset, and a 100 ms silent period was added after the end of the utterance to provide stable ending transitions.

A pilot fMRI study (see infra) aided us in determining the low-level auditory features that had to be controlled for, such as the duration and sound level, which very much determined responses in early auditory areas. Stimuli can be found here: https://osf.io/t7xp9/.

### Experimental fMRI Runs

The fMRI session consisted of six runs, each with 96 pseudo-randomly presented experimental trials, i.e., all 12 visual stimuli paired with all eight auditory stimuli, to make the design orthogonal for each sensory dimension (see Figure 1). Additionally, 10 silent fixation trials were included among them, as well as three initial and three final dummy trials, making a total of 112 trials, with 4.5 s of Stimulus Onset Asynchrony (SOA), totalizing 504 s of duration per run. Each experimental trial started with a visual image for 2.5 s during which an auditory utterance was played via headphones (from 0.6 s to 1.3 s to simulate a natural delay before the vocal reaction. A 2-s fixation cross was then displayed until the end of the trial (see Figure 1B). Subjects could respond any time within the trial and were instructed to press the buttons as soon as they know the answer.

### Pilot fMRI Experiment

A pilot fMRI study was conducted with 13 subjects, using a very similar design. The main difference between the pilot and the actual experiment was that the pilot did not contain a counterbalancing of response button in terms of congruency, which made it impossible to dissociate congruency representations from motor responses. There were a few other more minor differences, such as the use of 24 instead of 12 visual stimuli, 4-button responses instead of two, and less stringent calibration of the auditory stimuli (not all stimuli had the same duration and RMS power). The pilot study was useful to determine and optimize several important aspects (design, analysis) for our main study in order to avoid circular analyses (Kriegeskorte et al., 2009) and will be mentioned at the relevant points in the manuscript.

### fMRI Data Acquisition

Imaging data were acquired using a 3T Philips Ingenia CX scanner (Department of Radiology of the University of Leuven) with a 32-channel head coil. Each functional run consisted of T2*-weighted echoplanar images (EPIs), with voxel size = 2.52 × 2.58 × 2.5, interslice gap 0.2 mm, TR = 2550 ms, TE = 30 ms, matrix = 84 × 82, 45 slices, field of view (FOV) = 211 × 211 × 121. In addition to the functional images we collected a high-resolution T1-weighted anatomical scan for each participant (182 slices, voxel size = 0.98 × 0.98 × 1.2 mm, TR = 9.6 ms, TE = 4.6 ms, 256 × 256 acquisition matrix). Stimuli were presented using Psychtoolbox 3 (Brainard, 1997). Visual stimuli were displayed via an NEC projector with a NP21LP lamp that projected the image on a screen the participant viewed through a mirror. Viewing distance was approximately 64 cm. Auditory stimuli were presented through headphones at a comfortable hearing level.

### fMRI Preprocessing

Imaging data were preprocessed and analyzed using the Statistical Parametrical Mapping software package (SPM 8, Welcome Department of Cognitive Neurology, London, UK) and MATLAB. Functional images underwent slice timing correction (ascending order; first image as reference), motion correction (3rd degree spline interpolation), co-registration (anatomical to functional images; mean functional image as reference), and spatial normalization to the standard Montreal Neurological Institute (MNI) brain space. Functional images were resampled to a voxel size of 2.2 × 2.2 × 2.7 mm and spatially smoothed by convolution of a Gaussian kernel of 5 mm full-width at half-maximum (Op de Beeck, 2010). One run of one subject was not considered due to excessive head movement.

### General Linear Models (GLMs)

We analyzed the fMRI data through GLMs. For each participant and run, pre-processed images were modeled for each voxel. They included regressors for each experimental condition and the six motion correction parameters (x, y, z for translation and rotation). Each predictor’s time course was convolved with the canonical hemodynamic response function (HRF) in SPM. In order to analyze the three manipulated dimensions (i.e., visual, auditory and social congruency), we built three separate GLMs. First, in the visual GLM, the 12 visual stimuli were declared as conditions, with onsets and duration corresponding to the visual presentation time (i.e., from 0 s to 2.5 s. Second, the auditory GLM contained the eight auditory stimulus conditions, with the corresponding auditory presentation time (from 0.6 s to 1.3 s. Third, the social congruency GLM had two conditions (congruent vs. incongruent), modeled from the beginning of the auditory presentation (0.6 s from trial onset) until the end of the trial (4.5 s. The detailed results of the social congruency GLM are reported in full elsewhere (Pegado et al., 2017) but we still present here the essential information of methods and results for the sake of completeness.

Note that* a priori* we considered two possible approaches to analyze the data, the here described approach with three separate GLMs (visual, auditory, congruency), and a “full-GLM” modeling approach with the 96 Audio-Visual (A-V) combinations of conditions, with each trial modeled as an event. We tried both approaches on the data from the pilot experiment. Although both approaches provided a very similar pattern of results, the full-GLM approach seemed to result in less stable results with a lower Signal-to-Noise Ratio (SNR), possibly because each A-V combination only had a single presentation per run. Moreover, by creating separate GLMs we could use more precise onsets and durations of each processing involved (visual, auditory and social congruency processing). Based on these considerations, the main experiment was analyzed with three separate GLMs.

### Regions of Interest (ROIs)

**Figure 2:**
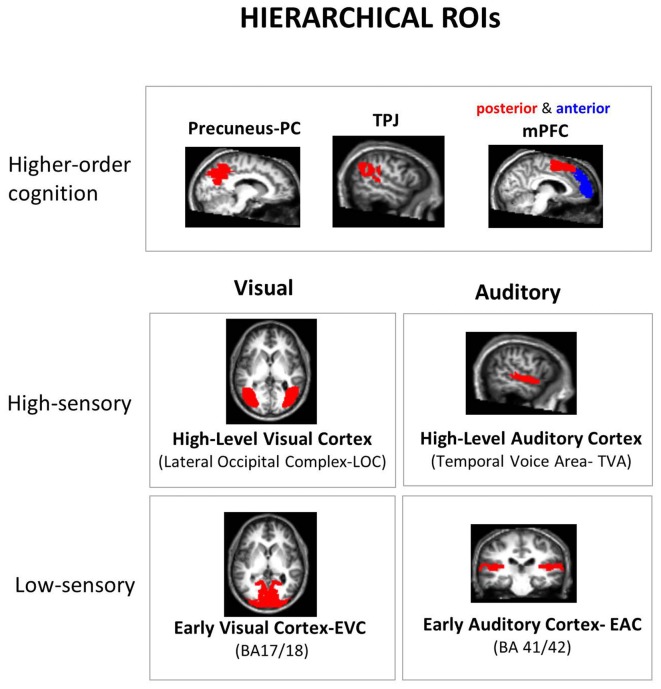
Hierarchical regions of interest (ROI). *A priori* ROIs were selected according to the hierarchical organization of the neural systems underpinning the processing of the manipulated dimensions: low and high-level sensory areas in each sensory modality and higher-order “mentalizing network” areas. All ROIs were defined using independent masks, either from anatomical atlas or functional parcels from other laboratories: Broadman’s Areas (BA) for low-level sensory areas and Precuneus (PC), “Temporal Voice Areas” (TVA) from Belin’s lab (Pernet et al., 2015; http://neurovault.org/collections/33/), Lateral Occipital Cortex (LOC) from Kanwinsher’s lab (Julian et al., 2012), and parcels from functional connectivity studies for medial Prefrontal Cortex (mPFC; anterior vs. posterior mPFC: amPFC vs. pmPFC; Sallet et al., 2013) and Temporo-Parietal Junction (TPJ; Mars et al., 2011; only right-hemisphere parcels are available for the latter two ROIs). The final subject-specific ROIs were made by a conjunction of these predefined ROI masks with all the active voxels (details in “Materials and Methods” section).

As regions of interest (ROI) analysis can be more sensitive than searchlight analysis (Bulthé et al., 2014), *à priori* ROIs were selected based on the literature, restricted to key brain regions involved in visual, auditory and social inference processing. While taking into account the hierarchical organization of these three neural systems, we included low- and high-level visual and auditory areas, and a higher-order mentalizing network (Figure 2). Our approach was to select the best available templates to delineate brain areas corresponding to each level of processing in each neural system, avoiding in that way both manual delineation of ROIs and the use of several functional localizers. For brain regions where anatomical landmarks are known to provide a proper approximation of functional regions (e.g., Brodmanns’ area BA 17 and 18 for early visual cortex, EVC), anatomical masks were used, from the anatomical atlas WFU PickAtlas Toolbox (Wake Forrest University PickAtlas^1^). For other regions where pure anatomical delimitation were less appropriate than functionally-defined regions, e.g., Lateral Occipital Complex (LOC) for high-level visual processing (Julian et al., 2012), we used parcels obtained by other laboratories (see details below). These general masks were combined (by means of a conjunction analysis) with individual functional data that specify voxels modulated by our task: the F-contrast of all task trials against fixation trials, at a threshold of 0.0001 (uncorrected for multiple comparisons), using a separate “neutral GLM” where all task trials were modeled as a single condition (fixation was implicitly modeled). ROIs with at least 20 active voxels were included. If a given participant ROI did not meet these criteria, his/her data was not used in the group analysis for this ROI. This situation only took place for two subjects in the anterior mPFC.

For sensory processing, a *low level visual ROI* (Early Visual Cortex-EVC) was defined based on Brodmann’s areas (BA) 17 and 18 as they are widely accepted landmarks for low level visual processing. As a *high-level visual ROI*, a functional parcel of the LOC from the Kanwisher lab was used (Julian et al., 2012). The *low-level auditory ROI* (Early Auditory Cortex, EAC) was composed by BA 41 and 42. The *high-level auditory ROI* was based upon the “Temporal Voice Area”-TVA probabilistic map from Belin’s lab (Pernet et al., 2015) concerning more than 200 subjects, available at neurovault^2^. The two low-level ROIs (EVC and EAC) presented very thin configurations. This would lead to unrealistic delimitations of early processing cortex given the spatial uncertainty involved when comparing brains across subjects. We thus made them thicker by 1 voxel in all three directions. This procedure is nowadays already incorporated in PickAtlas through the 3D dilatation function. For *higher-order cognition ROIs*, since we used a mentalizing task we targeted the mentalizing neural network: mPFC, TPJ and PC. Note that these regions are also known to process representations of emotions and valence in both visual and auditory modalities (Peelen et al., 2010; Klasen et al., 2011). Following the same approach as a recent meta-analysis of different mentalizing tasks (Schurz et al., 2014), we used the same parcels that were obtained in functional connectivity studies, both for mPFC (Sallet et al., 2013) and TPJ (Mars et al., 2011). Note that only right hemisphere parcels are available for these two ROIs. Further, as we did not have particular hypotheses for the two subdivisions of TPJ (anterior and posterior parcels) we grouped them together in a single ROI. In contrast, for the mPFC, we kept this distinction as the literature shows a clear functional dissociation between anterior vs. posterior parts for self-related vs. others-related mentalizing processes respectively (Mitchell et al., 2006; Saxe et al., 2006; Denny et al., 2012; Sul et al., 2015), by integrating the four original parcels into two. Finally, for PC, we used the anatomical mask in WFU Pickatlas (SPM). Given its role in emotional processing, an amygdala ROI was initially included (using anatomical atlas) in the analysis of the pilot fMRI experiment. Yet, as it did not show detectable information in any dimension, it was not included here.

To ensure that no overlap occurs between ROIs, we visually inspect ROI borders of each ROI pair. As a first measure, we restricted the TVA probabilistic map to the most significant voxels by imposing an arbitrary threshold of *t* = 50, which restricted the ROI to their classical temporal cortex disposition and reduced considerably its overlap with other regions (e.g., TPJ). The resulting map was then transformed in a binary mask. As an additional measure, for this and all the other ROIs (all binary masks), we excluded the remaining overlapping voxels from the largest ROI of each pair. Only the following ROI intersections presented some overlap: EVC × LOC, EVC × PC, EAC × TVA, EAC × TPJ and TVA × TPJ (the first of each pair being the largest one). This procedure ensured a complete separation of the ROIs, without “denaturing” their original spatial configuration.

### Correlation-Based Multivoxel Pattern Analysis

We used correlation-based multivoxel pattern analysis (MVPA) to explore how the spatial response pattern in individual ROIs differs between experimental conditions (Haxby et al., 2001). For each GLM and participant, we extracted the parameter estimates (betas) for each condition (relative to baseline) in each voxel of the ROIs. These obtained values for each run were then normalized by subtracting the mean response across all conditions (for each voxel and run separately), to emphasize the relative contribution of each condition beyond global activation level, as previously done in the literature (Op de Beeck et al., 2008; Bracci and Op de Beeck, 2016). The full dataset (six runs) were randomly divided into two independent subsets of runs (using “randperm” function in Matlab). Thus, typically three runs were randomly assigned set 1 and three other runs to the set 2 of the classification procedure. In the single case of incomplete data (five runs instead of six), only two runs were assigned as set 1. The multi-voxel patterns of activity associated with each condition in set 1 (runs averaged) were pairwisely correlated with the activity patterns in set 2 (runs averaged) by using the “corrcoef” function in Matlab (Persons’ *r* correlation coefficient). This procedure of splitting the data in two parts followed by correlating the multi-voxel patterns was repeated 100 times. The final neural similarity matrix for each ROI was obtained by averaging these 100 matrices. Representational Similarity Analysis (RSA; Kriegeskorte et al., 2008a) could then be used to estimate informational content in the neural matrices. The matrix size varied as a function of the GLM: 12 × 12 for visual, 8 × 8 for auditory and 2 × 2 for social congruency GLMs.

### MVPA Pipeline

We adopted a hierarchical step-by-step approach to analyze the data to address three successive questions: (1) Which regions contain information of a given dimension? (2) What kind of representation do they contain? (3) How much information is shared with other regions?

#### Selection of Informative ROIs

To test whether a certain region contained information about a given dimension (visual, auditory, or social congruency), we applied the following procedure. First, we calculated for each ROI, the mean correlations in the diagonal and non-diagonal cells of the neural similarity matrix. Then, we performed a two sample two-tailed *t*-test across participants for diagonal vs. non-diagonal mean correlations. This procedure is based on the fact that the same condition will typically show higher similarity across runs relative to different conditions (i.e., higher correlations for diagonal vs. non-diagonal cells (Ritchie et al., 2017). Lastly, a Bonferroni correction for multiple comparisons (i.e., the number of ROIs) was applied.

#### Comparing Neural Representations With Theoretical Models of Visual and Auditory Processing

For each ROI containing information of a given dimension (i.e., significant *t*-test in the previous step), we compared the neural similarity matrices with similarity matrices of explicit models, to understand the kind of representational content of each ROI. In order to avoid the influence of the correlation of each stimulus with itself (diagonal cells) we only included the non-diagonal cells, averaging upper and lower triangles of the neural similarity matrix. This final vector was used in all further representational analysis. Each theoretically-relevant model targeted a specific parameter of the stimulus set. The values of each condition were compared pairwise with all the other conditions to establish the similarity between each pair, leading to 12 × 12 visual, 8 × 8 auditory and 2 × 2 social congruency similarity matrices.

##### Visual models

Four visual models were included for the RSA in this study. (1) a pixelwise model: based on pixel values of each visual stimulus; (2) a luminance model: based on mean luminance values; (3) a visual valence model: based on group average ratings of valence (1–9 level) obtained outside the scanner; and (4) an animacy model: a binary model based on animacy of images (animate vs. inanimate).

##### Auditory models

We also included four auditory models for RSA purposes. (1) a fundamental frequency (F0) model: based on fundamental frequency of audio stimuli; (2) an auditory valence model: based on group valence ratings obtained outside the scanner; (3) an utterance emotion model: based on the four emotions conveyed by the utterances (fear, disgust, admiration and cuteness-like expressions); and (4) a voice gender model: based on the gender of the voice.

**Figure 3:**
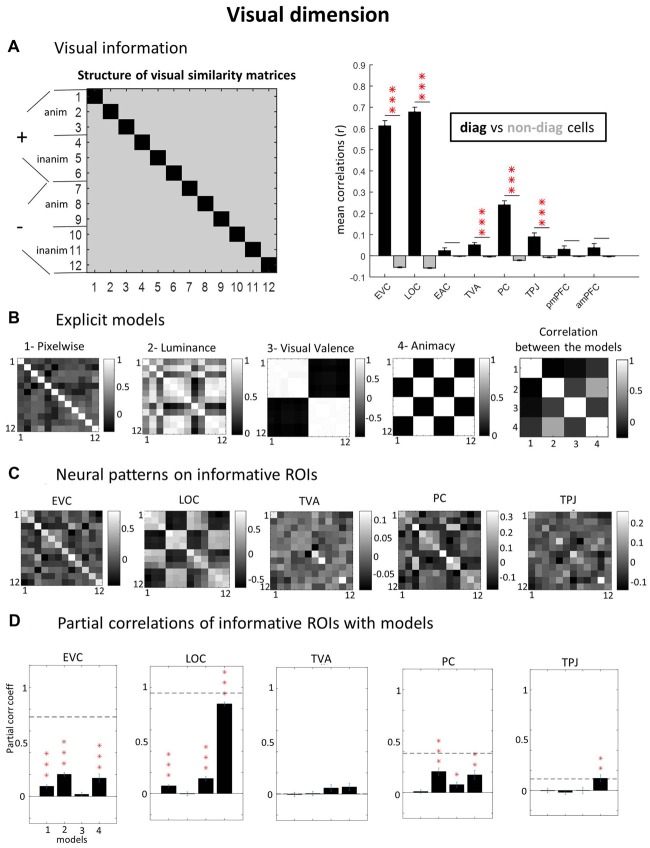
Visual dimension. **(A)** ROIs with visual information (diagonal vs. non-diagonal). Using a “visual GLM” where only the 12 visual conditions are declared and applying correlation-based Multivoxel Pattern Analysis (MVPA), we calculated neural similarity matrices for each subject and ROI (left panel). To verify which ROI contain visual information, we contrasted pair of trials with the same visual image (diagonal cells, in black) against pairs of trials with different images (non-diagonal cells, in gray). If particular visual information would be present in the ROI, the correlations in the diagonal cells would be significantly higher than in non-diagonal cells. **(B)** Explicit models. To investigate the kind of visual representation present in each informative ROI we built explicit models in relation to specific stimulus parameters: pixel-by-pixel values, mean luminance, group average visual valence ratings and animacy (animate vs. inanimate images). We also calculated the correlation among the models (extreme right). **(C)** Neural patterns on informative ROIs. Group average neural similarity matrices for each ROI are plotted. **(D)** Partial correlations of informative ROIs with models. To test the best predictive models of neural patterns, we performed partial correlations of neural and model matrices (using only non-diagonal cells, averaged across upper and lower triangles). Horizontal dashed lines represent Signal-to-Noise Ratio (SNR) estimations (see “Materials and Methods” section). Note that the SNR for TVA is close to zero (5.6e-04) and cannot be visualized. *P*-values of paired *t*-tests across subjects were Bonferroni-corrected, considering the number of ROIs, leading to the following thresholds: *p < 0.0063; **p < 0.0013; ***p < 1.25e-04. Error bars = ± SEM.

**Figure 4:**
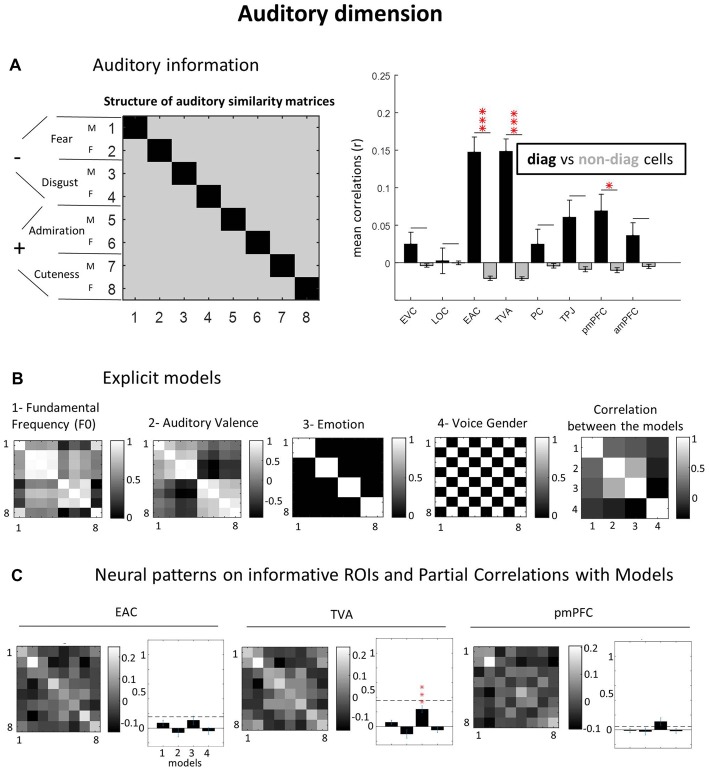
Auditory dimension. **(A)** Auditory information. **(B)** Explicit models. **(C)** Neural patterns on informative ROIs and partial correlations with models. As for the visual domain, we created for the auditory dimension, an “auditory GLM” declaring the eight auditory conditions, and then applying correlation-based MVPA. Models were built based on Fundamental Frequency (F0), group average auditory valence ratings, emotional content (fear, disgust, admiration and cuteness like) and voice gender. See caption of Figure 3 for equivalent explanation (**C,D** panels are collapsed here). *p = 0.05; ***p = 0.001 (Bonferroni-corrected).

To capture the specific contribution of each model explaining the neural pattern, we used partial correlation analysis, instead of simple correlations (testing the significance of coefficient estimates with *t*-tests against zero). This is particularly important when some models correlate with each other. Additionally, a neural SNR was estimated based on the correlation of each participant’s neural representational matrix (averaged over upper and lower triangle of non-diagonal cells) with all the other participant’s matrices averaged (Bracci and Op de Beeck, 2016). The average of these correlations across all subjects is represented by dashed lines in Figures 3D, 4C.

#### Investigating Information Shared Across ROIs (2nd Order Correlations)

Finally, we investigated how information was shared across brain regions. For each ROI, we concatenated the matrix values of the three manipulated dimensions into a single vector, using only the non-diagonal cells (Figure Figure 6A). In order to compensate for differences in the number of vector elements from the three dimensions, we used a weighting procedure: the values from the smaller matrices were multiplied by the square root of the number of observations in the largest dimension, i.e., in the visual dimension (66 observations), divided by the number of observations in the smaller dimension. The results obtained are equivalent of those produced by multiplying the values of each dimension or by replicating the cells for each dimension as many times as the Least Common Multiple (LCM). Then, we used this concatenated vector to test how the information pattern contained in each ROI correlated to those in the other ROIs, tested in a pairwise way (Figure 6B). Complementary, we also performed Multidimensional Scaling (MDS) to check how these ROIs cluster together when using the full informational concatenated vector. The MDS algorithm (“mdscale” function in Matlab) received as input the dissimilarity between the ROIs computed as 1 minus the correlation between vectors as shown in Figure 6A. All the pairwise dissimilarities between ROIs result in the matrix shown in Figure 6B. We used 1000 iterations, as it was a sufficient number for reliable results, to avoid the influence of the random initial values of the iterative fitting procedure used in MDS (indeed, even 100 repetitions provided already very stable results). To obtain a visual estimation of the relative contribution of each dimension in each brain region, we plotted pies instead of dots in the MDS figure, using the diagonal minus non-diagonal values in each dimension normalized to create the relative proportions in the pie. The pies were scaled using the sum of these three values. Thus, a region with large differences between diagonal and non-diagonal will have a large pie (Figure 6C). Note that non-diagonal cells in neural matrices can have significant information of a given dimension (e.g., visual), specially to *qualitatively* characterize the type of representation (visual animacy). However, for a *quantitative* estimation of information in that dimension, we used the diagonal minus non-diagonal values, as correlations for the same condition across runs can be expected to be higher than for different conditions, on average, if the ROI cares about that dimension. In other words, the difference between diagonal vs. non-diagonal cells indicates the *level* of information sensitivity on a given dimension, without entering in the consideration of what *kind* of representation.

**Figure 5:**
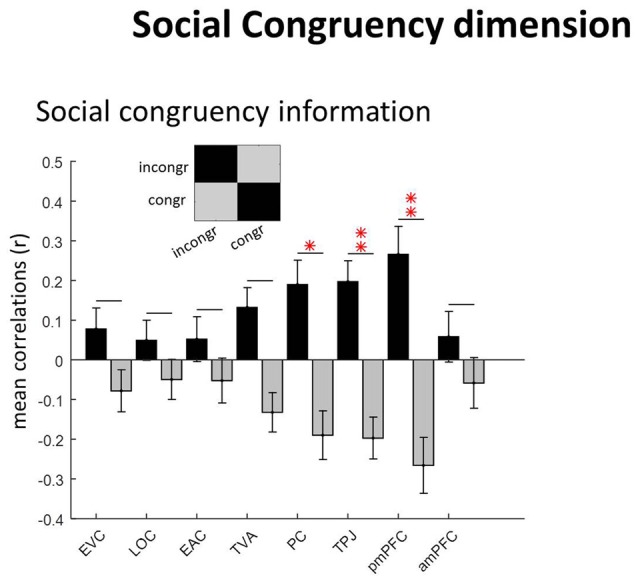
Social congruency dimension. The “social congruency” GLM had two conditions: congruent and incongruent. ROIs with social congruency information (diagonal vs. non-diagonal; see caption of Figure 3 for equivalent information). *p = 0.05.

**Figure 6:**
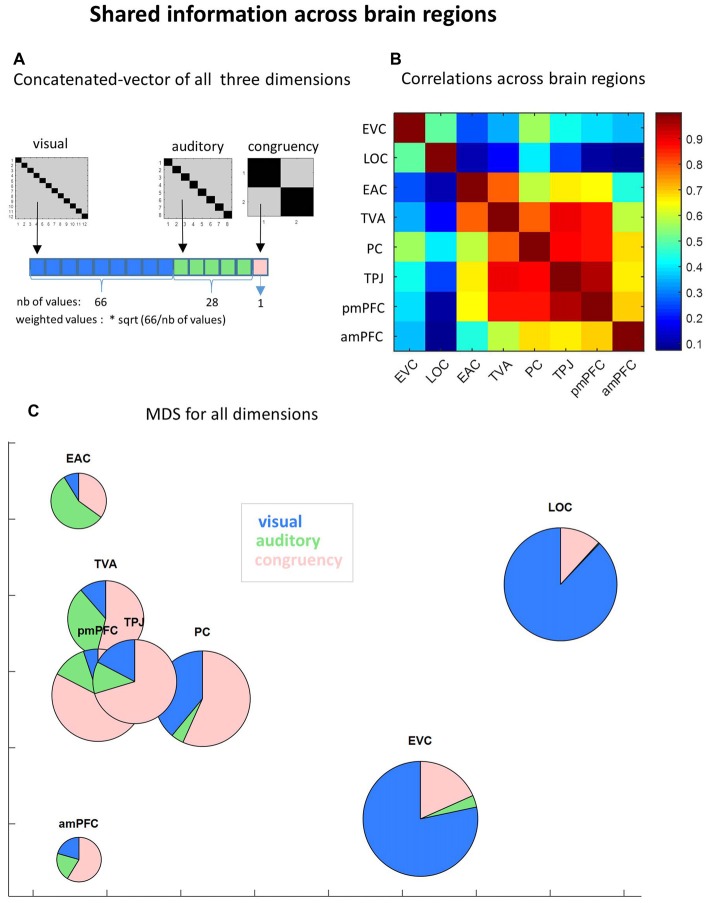
Shared information across brain regions.**(A)** Concatenated vector of all three dimensions. To evaluate how information was shared across brain regions, we built a concatenated vector from the neural similarity matrices of the three manipulated dimensions (visual, auditory and congruency), using only non-diagonal cells (upper and lower triangles averaged). Each dimension vector was weighted to compensate for the different number of values. **(B)** Correlation across brain regions. Pairwise Pearson correlations across brain regions were performed using the group average concatenated vector. **(C)** MultiDimesional Scaling (MDS) for all dimensions. MDS was applied (1000 iterations) to the same concatenated vectors to verify the clustering of ROIs based on common information. To visualize the specific informational content of each ROI we plotted pies instead of dots, showing the relative contribution of each dimension (diagonal minus non-diagonal). Pies are scaled in proportion to the total amount of information (sum of diagonal minus non-diagonal in all experimental dimensions).

### Searchlight MVPA Analysis

Searchlight MVPA analysis was used as a complementary way to check for potential missing anatomical areas outside our* a priori* ROIs, notably for unisensory valences, a feature that we could not capture well in our ROIs (significant but small correlations for visual valence, no correlations for auditory valence). We used “cosmo MVPA” searchlight scripts (Oosterhof et al., 2016) to perform individual analysis on each separate dimension, using the default parameters (e.g., spherical neighborhood of 100 voxels). The searchlight results were smoothed to 8 mm full-width at half-maximum and a 2nd level analysis was performed (both using SPM). One participant was excluded from this analysis because of a missing run (excluded for excessive movement). No extra brain region captured unisensory valence or the other studied dimensions.

### Univariate Analysis

Correlations between activity patterns (MVPA) are not necessarily related to overall differences in activity level between conditions (univariate analysis). Nevertheless, it is relevant to know whether MVPA findings are found in the context of effects that can also be picked up by a univariate voxel-level difference in activity (FWE corrected at *p* < 0.05). One distinction was very obvious in a univariate analysis, namely animate vs. inanimate visual pictures, which is to be expected given the extensive literature on category selectivity. Interestingly, we could observe univariate effects when contrasting negative minus positive images in high-level visual areas (LOC and ventral temporal cortex; peak at 31, −60, −13), but not in the opposite direction, which can be potentially linked to a relative attentional enhancement provoked by negative images. Other tested distinctions failed to reveal significance in a whole-brain univariate analysis. The limited sensitivity of the univariate analyses is overcome by correlational MVPA which allows us to capture the richness of multiple neural representations.

### Data Availability

The files needed to replicate the analyses (e.g., ROI definitions and individual subject representational similarity matrices) are available on the Open Science Framework website^3^. Other aspects of the data (e.g., raw data files, other steps in the analyses) are available from the corresponding author on reasonable request.

## Results

We will first report the results from the visual dimension (low and high-level visual representations), then those of the auditory dimension (low and high-level auditory representations), followed by the results concerning the congruency between these two dimensions (social congruency representations). Finally, we will report the similarities of neural representations between brain areas, by using the informational content from all three dimensions at once.

### Low- and High-Level Visual Representations

First, to determine which ROI represented visual information, we compared the diagonal vs. non-diagonal cells in visual GLM neural similarity matrices (see “Materials and Methods” section). This analysis revealed several regions with significant visual representations (Figure 3A): EVC (*r* = 0.61 vs. −0.05; *p* < 0.0001), LOC (*r* = 0.68 vs. −0.06, *p* < 0.0001), TVA (*r* = 0.05 vs. −0.005, *p* = 0.0009), PC- PC (*r* = 0.24 vs. −0.02; *p* < 0.0001) and TPJ (*r* = 0.09 vs. −0.01, *p* = 0.0007). All *t*-tests comparing the consistency of MVPA representations in diagonal vs. non-diagonal cells’ were Bonferroni-corrected for the number of ROIs tested and results were considered significant if bellow *p* = 0.05 after correction;all reported *p-values* in the text are corrected). Other regions did not show significant results: EAC (*r* = 0.02 vs. −0.002, *p* = 0.66), posterior mPFC (*r* = 0.03 vs. −0.003; *p* = 0.60) and anterior mPFC (*r* = 0.04 vs. −0.003; *p* = 0.77).

Next, we determined what types of visual representations were captured in the different ROIs. Therefore, explicit visual models (Figure 3B) were compared to the neural similarity matrices (Figure 3C) to characterize the kind of representation in visually-informative ROIs, using Pearsons’s partial correlations (Figure 3B). EVC data was significantly explained by the low-level feature models for pixels (correlation coefficient *r* = 0.09; *p* < 0.0001) and luminance (*r* = 0.20; *p* = 0.0009) but also by the high-level animacy model (*r* = 0.17; *p* = 0.0002), while no effect was found for the visual valence model (*r* = −0.02; *p* > 0.9). All partial correlations were Bonferroni-corrected for the number of models tested. LOC data was most strongly explained by the animacy model (*r* = 0.85; *p* < 0.0001), confirming that high-level visual cortex (LOC) made a clear distinction between animate vs. inanimate images. The luminance model showed non-significant results in LOC (*r* = −0.003; *p* > 0.9). As it partially co-varied with the animacy model, the luminance model could have been overshadowed by the substantial amount of variance explained by the animacy model. Further, LOC also represented low level pixelwise features (*r* = 0.07; *p* < 0.0001) and visual valence (*r* = 0.14; *p* < 0.0001). PC showed positive partial correlations for luminance (*r* = 0.20; *p* < 0.0001), valence (*r* = 0.08; *p* = 0.02) and animacy (*r* = 0.17; *p* = 0.003), but not for the pixelwise (*r* = 0.01; *p* > 0.9) model. TPJ data was significantly explained by the animacy model (*r* = 0.12; *p* = 0.008), but not by the other models (pixelwise: *r* = −0.004; *p* > 0.9; luminance: *r* = −0.02; *p* > 0.9; valence: *r* = 0.001; *p* > 0.9). Finally, none of our visual models captured the kind of visual information represented in TVA (pixelwise: *r* = −0.005; *p* > 0.9; luminance: *r* = 0.004; *p* > 0.9; valence: *r* = 0.06; *p* = 0.41; animacy: *r* = 0.07; *p* = 0.28). Note that the SNR in this region (horizontal dashed line) for the visual dimension was very low.

### Low- and High-Level Auditory Representations

As for the visual dimension, we first determined which brain regions contained auditory information by performing a correlational MVPA. Three regions hosted significantly distinguishable auditory representations: EAC (*r* = 0.15 vs. −0.02; *p* < 0.0001), TVA (*r* = 0.15 vs. −0.02; *p* < 0.0001) and posterior mPFC (*r* = 0.07 vs. −0.01; *p* = 0.045). For the other ROIs, we did not observe auditory information: EVC (*r* = 0.02 vs. −0.003; *p* > 0.9), LOC (*r* = 0.003 vs. −0.0002; *p* > 0.9), PC (*r* = 0.03 vs. −0.004; *p* > 0.9), TPJ (*r* = 0.06 vs. −0.01; *p* = 0.13) and anterior mPFC (*r* = 0.03 vs. −0.005; *p* = 0.52; see Figure 4A).

Partial correlations with the explicit auditory models (Figure 4B) revealed that only the “utterance emotion” model significantly explained TVA data (*r* = 0.24; *p* < 0.0001), in contrast to the other models which did not demonstrate this effect (F0: *r* = 0.06; *p* = 0.19; auditory valence: *r* = −0.1; *p* = 0.36; voice gender: *r* = −0.05; *p* = 0.81 (see Figure 4C). A similar pattern was found for EAC, however it did not reach significance level for any of the models (F0: *r* = 0.07; *p* = 0.30; auditory valence: *r* = −0.06; *p* > 0.9; utterance emotion: *r* = 0.11; *p* = 0.29; voice gender: *r* = −0.04; *p* > 0.9). Also for pmPFC, utterance emotion was the model explaining most of the variance but did not reach significance with correction for multiple comparisons: F0: *r* = −0.01; *p* > 0.9; auditory valence: *r* = −0.02; *p* > 0.9; utterance emotion: *r* = 0.11; *p* = 0.16; voice gender: *r* = −0.01; *p* > 0.9. Note the lower SNR (dashed horizontal lines) for both EAC and pmPFC relative to TVA. It is worth noting that we did quite some effort to equate stimuli on various low-level dimensions. We did not do this to the same extent in the pilot experiment, where stimuli varied in sound duration and RMS power, which resulted in much stronger auditory representations in EAC.

### Representations of Social Congruency

These results have been reported in full elsewhere (Pegado et al., 2017). Consistent with the analysis of the previous dimensions, we again used the diagonal vs. non-diagonal approach to determine the regions hosting social congruency information (Figure 5). As expected, regions of the neural mentalizing network host significant informational content: PC (*p* = 0.046), TPJ (*p* = 0.01) and posterior mPFC (*p* = 0.009). However, this was not the case for the anterior mPFC (*p* > 0.9) nor for any of the other sensory regions: EVC (*p* > 0.9), LOC (*p* > 0.9), EAC (*p* > 0.9), TVA (*p* = 0.12; Bonferroni-corrected as previously). Note that for two participants no active clusters were even found in the anterior mPFC.

### Shared Information Across Brain Regions

In order to uncover the differences and similarities between brain regions in what they represent and what not, we concatenated non-diagonal cells of similarity neural matrices of the three manipulated dimensions (Figure 6A) and then tested how the global informational content was shared among brain regions. First, by running pairwise correlations between ROIs with the full informational vector, a dissociation between the two sensory systems was noticed: high correlations among visual areas on the one hand (*r* = 0.50; *p* < 0.0001) and among auditory areas on the other hand (*r* = 0.80; *p* < 0.0001) but very low correlations between different sensory modalities (see Figure 6B). Further, PC shares information with visual areas, especially with EVC (*r* = 0.56), and TPJ with auditory areas (with EAC: *r* = 0.66; with TVA: *r* = 0.89; all *p* < 0.0001), a pattern that follows anatomical distance. Also following the anatomical distance principle, anterior and posterior mPFC showed high correlation between them (*r* = 0.69, *p* < 0.00001), despite previously observed differences in social information content (see Figure 5). This is important to ensure that informational content detected in anterior mPFC is not merely random noise and that differences of social information between posterior and anterior mPFC can be meaningful.

Besides anatomical proximity, functionally related distant regions also presented similarity of neural representations. Areas of the mentalizing network show high information similarity: PC × TPJ: *r* = 0.88; PC × posterior mPFC: *r* = 0.86; PC × anterior mPFC: *r* = 0.67; TPJ × posterior mPFC: *r* = 0.95; TPJ × anterior mPFC: *r* = 0.67; all *p* < 0.0001). Finally, TVA also show similar patterns of information content relative to mentalizing network areas: TVA × PC: *r* = 0.80; TVA × TPJ: *r* = 0.89; TVA × posterior mPFC: *r* = 0.87; and TVA × anterior mPFC: *r* = 0.59; all *p* < 0.0001.

MDS analysis using the concatenated vector illustrates these informational similarities between the ROIs (Figure 6C). By using scaled pies instead of dots, we illustrate the differences between ROIs in their specific (percentage on each dimension) and total (size) informational content.

## Discussion

To shed light upon how the human brain performs complex tasks requiring integration of multiple information as typically performed in natural social environments, we investigated brain representations at different hierarchical levels and neural systems, all at once (i.e., within the same trial) by using an audio-visual social perception paradigm. We manipulated the visual, auditory and social processing dimensions in an orthogonal way, aiming to obtain a more holistic view of how the brain process these multiple levels of information together. We showed already before that “social norm” inferences are processed in the brain regions involved in theory of mind judgments, the so-called mentalizing network: PC, TPJ and posterior (but not anterior) mPFC. In the same data we find characteristic hierarchical neural patterns in the visual and auditory systems. By analyzing these representations together we could observe that: (1) brain regions clearly differed in the representations that they host as no two regions of interest represented the same combination of features; (2) brain regions could host multiple types of representations, either within one dimension (e.g., EVC represents luminance, pixel value and animacy status of the images), or across dimensions (e.g., see representations of TVA or of the mentalizing network in Figure 6); and (3) robustness of neural representations is not determined by task-relevance, e.g., high-level visual cortex (LOC) strongly distinguishes animate from inanimate pictures despite its task-irrelevance, while task-relevant auditory valences were much weaker.

Our approach provides a rich view of the brain representational architecture. In the visual domain, we confirmed the processing hierarchy, albeit with overlap between low and high-level visual cortex representations. We noticed that EVC represents several low-level visual features (Figure 3D), as well as higher level animacy distinction. In line with the literature (Kriegeskorte et al., 2008b; Bracci and Op de Beeck, 2016), this animate vs. inanimate distinction was much stronger in LOC (Figures 3C,D), but importantly, we also observed here representations of low-level features (pixel values), as has recently been high-lighted (Hong et al., 2016). In addition, LOC showed a significant representation of visual valence.

In the auditory domain, the EAC showed significant sensitivity to auditory information. However, the exact representations could not be explained by any of the low-level auditory models. Indeed, as we have a small number of stimuli, we tried to control low-level parameters, such as power and duration, especially because in our pilot fMRI study, EAC responses were massively driven by variations in stimulus duration. Previous research has demonstrated the capacity of MVPA to decode basic non-verbal utterance information in the auditory cortex, such as vowels (Formisano et al., 2008). Here, we could confirm that TVA can differentiate emotional content in vocal utterances (Figure 4C) as previously reported (Ethofer et al., 2009).

Further, social norm inference information was found in all three ROIs of the core mentalizing network (Amodio and Frith, 2006; Frith and Frith, 2006; Mitchell, 2009; Schurz et al., 2014): PC, TPJ and mPFC (in posterior but not in anterior part; see Figure 5). Given that we used an allocentric perspective task during the scanning, which was validated behaviorally as described in Pegado et al. (2017), these results are in accordance with previous works showing a dissociation between anterior vs. posterior mPFC for egocentric vs. allocentric mentalizing processes respectively (Mitchell et al., 2006; Saxe et al., 2006; Denny et al., 2012; Sul et al., 2015) and further extends this notion for a quite abstract (instead of concrete) “other” used as the mentalizing target, i.e., “most of people.”

Interestingly, our approach could provide a more nuanced picture of brain representations. For instance, visual and auditory information were not exclusively found in the two visual and the two auditory ROIs, respectively. TVA, PC and TPJ also represent visual information. Despite our efforts to constrain the regions of interest (see “Materials and Methods” section), we cannot exclude the possibility that part of the signal would originate from nearby (visual) regions that, at the group level, overlap with PC and TPJ. Further, even if normalization is perfectly performed, the limited resolution of fMRI could still generate overlap, as shown by Schwarzlose et al. (2005) that one needs high resolution to (partially) distinguish between body and face selectivite regions. Importantly however, our approach enables us to verify that regions such as TVA, PC and TPJ present a distinct overall functional profile of information relative to visual regions (Figures 6B,C). There are other examples where some regions show high levels of information outside the expected dimension. For instance, TVA seems to contain representations that go beyond auditory information. Interestingly, in the correlations between brain areas using the full informational vector (Figure 6B), TVA showed high similarity with other regions of the mentalizing network, despite the fact that TVA only showed a marginally significant effect of social congruency. This observation illustrates the added value of this 2nd-order similarity analysis (Figure 6) beyond the classical unidimensional threshold-based one.

In addition, the naturalistic multisensory social scenario adopted here sheds light on how task-relevance influenced the strength of neural representations across multiple systems. Our results suggest that task-relevance is not very critical. Indeed, the task-irrelevant animacy distinction in LOC was very robust (Figures 3C,D). In contrast, task-relevant features such as auditory valence could not be detected in any of the ROIs, and both visual and auditory valence could not be detected with a whole-brain searchlight analysis. A possible explanation is that valence and emotion can be represented in a very distributed way across brain regions (for recent studies and meta-analysis see Wager et al., 2015; Kragel and LaBar, 2016; Lindquist et al., 2016; Saarimäki et al., 2016). Another possibility is that one or more of the higher-order ROIs (mPFC, TPJ and PC) would represent valence in a supramodal way (Peelen et al., 2010; Klasen et al., 2011). Thus, valence in one sensory modality could have suffered a direct interference of the valence signal from the other modality, and in this case only valence congruency across-modalities (Klasen et al., 2011) would be detectable.

In conclusion, the present study illustrates the possibilities of RSA-RSA (Kriegeskorte et al., 2008a) by exploring multiple brain representations simultaneously, and in a more ecologically valid set-up. Future studies could benefit from such multi-level perspective, for instance, in multisensory research where hierarchical levels of each sensorial modality are rarely considered simultaneously. Further, the present strategy can also provide a different and more comprehensive perspective in domains where different processing levels are typically studied separately. The present strategy could for instance be particularly interesting to study sensory (low and high) and higher-order social processing in Autism Spectrum Disorders (ASD) aiming to verify potential differences relative to matched controls in each of these levels, all at once.

## Author Contributions

FP, BB and HOB designed the article. FP, MHAH, SA and ND performed the experiments. FP, JB, HLM and HOB analyzed the data. FP, JB, BB and HOB wrote the article.

## Conflict of Interest Statement

The authors declare that the research was conducted in the absence of any commercial or financial relationships that could be construed as a potential conflict of interest.

## Footnotes

^1^http://fmri.wfubmc.edu/cms/software

^2^http://neurovault.org/collections/33/

^3^https://osf.io/t7xp9/

## References

[B1] Amodio D. M. Frith C. D. (2006). Meeting of minds: the medial frontal cortex and social cognition. Nat. Rev. Neurosci. 7, 268–277. 10.1038/nrn1884 16552413

[B2] Bestelmeyer P. E. Maurage P. Rouger J. Latinus M. Belin P. (2014). Adaptation to vocal expressions reveals multistep perception of auditory emotion. J. Neurosci. 34, 8098–8105. 10.1523/jneurosci.4820-13.2014 24920615 PMC4051968

[B3] Bossens C. Op de Beeck H. P. (2016). Linear and non-linear visual feature learning in rat and humans. Front. Behav. Neurosci. 10:235. 10.3389/fnbeh.2016.00235 28066201 PMC5180255

[B4] Bracci S. Op de Beeck H. (2016). Dissociations and associations between shape and category representations in the two visual pathways. J. Neurosci. 36, 432–444. 10.1523/JNEUROSCI.2314-15.2016 26758835 PMC6602035

[B50] Brainard D. H. (1997). The psychophysics toolbox. Spat. Vis. 10, 433–436. 10.1163/156856897X00357 9176952

[B501] Bulthé J. van den Hurk J. Daniels N. Op de Beeck H. P. (2014). “A validation of a multi-spatial scale method for multivariate pattern analysis,” in Proceedings of the 4th International Workshop on Pattern Recognition in Neuroimaging (Tubingen: IEEE), 4–6. 10.1109/PRNI.2014.6858513

[B5] Dehaene S. Pegado F. Braga L. W. Ventura P. Nunes Filho G. Jobert A.. (2010). How learning to read changes the cortical networks for vision and language. Science 330, 1359–1364. 10.1126/science.1194140 21071632

[B6] Denny B. T. Kober H. Wager T. D. Ochsner K. N. (2012). A meta-analysis of functional neuroimaging studies of self- and other judgments reveals a spatial gradient for mentalizing in medial prefrontal cortex. J. Cogn. Neurosci. 24, 1742–1752. 10.1162/jocn_a_00233 22452556 PMC3806720

[B7] Ethofer T. Ville D. V. D. Scherer K. Vuilleumier P. (2009). Decoding of emotional information in voice-sensitive cortices. Curr. Biol. 19, 1028–1033. 10.1016/j.cub.2009.04.054 19446457

[B8] Formisano E. De Martino F. Bonte M. Goebel R. (2008). “Who” is saying “what”? Brain-based decoding of human voice and speech. Science 322, 970–973. 10.1126/science.1164318 18988858

[B9] Frith C. D. Frith U. (2006). The neural basis of mentalizing. Neuron 50, 531–534. 10.1016/j.neuron.2006.05.001 16701204

[B10] Haxby J. V. Gobbini M. I. Furey M. L. Ishai A. Schouten J. L. Pietrini P. (2001). Distributed and overlapping representations of faces and objects in ventral temporal cortex. Science 293, 2425–2430. 10.1126/science.1063736 11577229

[B11] Hong H. Yamins D. L. K. Majaj N. J. DiCarlo J. J. (2016). Explicit information for category-orthogonal object properties increases along the ventral stream. Nat. Neurosci. 19, 613–622. 10.1038/nn.4247 26900926

[B12] Julian J. B. Fedorenko E. Webster J. Kanwisher N. (2012). An algorithmic method for functionally defining regions of interest in the ventral visual pathway. Neuroimage 60, 2357–2364. 10.1016/j.neuroimage.2012.02.055 22398396

[B13] Kamitani Y. Tong F. (2005). Decoding the visual and subjective contents of the human brain. Nat. Neurosci. 8, 679–685. 10.1038/nn1444 15852014 PMC1808230

[B14] Kay K. N. Naselaris T. Prenger R. J. Gallant J. L. (2008). Identifying natural images from human brain activity. Nature 452, 352–355. 10.1038/nature06713 18322462 PMC3556484

[B15] Klasen M. Kenworthy C. A. Mathiak K. A. Kircher T. T. J. Mathiak K. (2011). Supramodal representation of emotions. J. Neurosci. 31, 13635–13643. 10.1523/jneurosci.2833-11.2011 21940454 PMC6623280

[B16] Kragel P. A. LaBar K. S. (2016). Decoding the nature of emotion in the brain. Trends Cogn. Sci. 20, 444–455. 10.1016/j.tics.2016.03.011 27133227 PMC4875847

[B17] Kriegeskorte N. Mur M. Bandettini P. (2008a). Representational similarity analysis—connecting the branches of systems neuroscience. Front. Syst. Neurosci. 2:4. 10.3389/neuro.06.004.2008 19104670 PMC2605405

[B18] Kriegeskorte N. Mur M. Ruff D. A. Kiani R. Bodurka J. Esteky H.. (2008b). Matching categorical object representations in inferior temporal cortex of man and monkey. Neuron 60, 1126–1141. 10.1016/j.neuron.2008.10.043 19109916 PMC3143574

[B19] Kriegeskorte N. Simmons W. K. Bellgowan P. S. F. Baker C. I. (2009). Circular analysis in systems neuroscience: the dangers of double dipping. Nat. Neurosci. 12, 535–540. 10.1038/nn.2303 19396166 PMC2841687

[B20] Lang P. Bradley M. Cuthbert B. (2008). International Affective Picture System (IAPS): Affective Ratings of Pictures and Instruction Manual. Gainsville, FL: University of Florida, Center for Research in Psychophysiology.

[B21] Lindquist K. A. Satpute A. B. Wager T. D. Weber J. Barrett L. F. (2016). The brain basis of positive and negative affect: evidence from a meta-analysis of the human neuroimaging literature. Cereb. Cortex 26, 1910–1922. 10.1093/cercor/bhv001 25631056 PMC4830281

[B22] Mars R. B. Jbabdi S. Sallet J. O’Reilly J. X. Croxson P. L. Olivier E.. (2011). Diffusion-weighted imaging tractography-based parcellation of the human parietal cortex and comparison with human and macaque resting-state functional connectivity. J. Neurosci. 31, 4087–4100. 10.1523/jneurosci.5102-10.2011 21411650 PMC3091022

[B23] McGurk H. MacDonald J. (1976). Hearing lips and seeing voices. Nature 264, 746–748. 10.1038/264746a0 1012311

[B24] Mitchell J. P. (2009). Inferences about mental states. Philos. Trans. R. Soc. Lond. B Biol. Sci. 364, 1309–1316. 10.1098/rstb.2008.0318 19528012 PMC2666715

[B25] Mitchell J. P. Macrae C. N. Banaji M. R. (2006). Dissociable medial prefrontal contributions to judgments of similar and dissimilar others. Neuron 50, 655–663. 10.1016/j.neuron.2006.03.040 16701214

[B26] Oosterhof N. N. Connolly A. C. Haxby J. V. (2016). CoSMoMVPA: multi-modal multivariate pattern analysis of neuroimaging data in matlab/GNU octave. Front. Neuroinform. 10:27. 10.3389/fninf.2016.00027 27499741 PMC4956688

[B27] Op de Beeck H. P. (2010). Against hyperacuity in brain reading: spatial smoothing does not hurt multivariate fMRI analyses? Neuroimage 49, 1943–1948. 10.1016/j.neuroimage.2009.02.047 19285144

[B28] Op de Beeck H. P. Torfs K. Wagemans J. (2008). Perceived shape similarity among unfamiliar objects and the organization of the human object vision pathway. J. Neurosci. 28, 10111–10123. 10.1523/JNEUROSCI.2511-08.2008 18829969 PMC6671279

[B29] Peelen M. V. Atkinson A. P. Vuilleumier P. (2010). Supramodal representations of perceived emotions in the human brain. J. Neurosci. 30, 10127–10134. 10.1523/JNEUROSCI.2161-10.2010 20668196 PMC6633378

[B500] Pegado F. Hendriks M. H. A. Amelynck S. Daniels N. Bulthe J. Lee Masson H.. (2017). Neural representations behind social norm inferences in humans. bioxriv 119. 10.1101/230508 30154471 PMC6113313

[B30] Pernet C. R. McAleer P. Latinus M. Gorgolewski K. J. Charest I. Bestelmeyer P. E. G.. (2015). The human voice areas: spatial organization and inter-individual variability in temporal and extra-temporal cortices. Neuroimage 119, 164–174. 10.1016/j.neuroimage.2015.06.050 26116964 PMC4768083

[B31] Pourtois G. de Gelder B. Bol A. Crommelinck M. (2005). Perception of facial expressions and voices and of their combination in the human brain. Cortex 41, 49–59. 10.1016/s0010-9452(08)70177-1 15633706

[B32] Ritchie J. B. Bracci S. Op de Beeck H. (2017). Avoiding illusory effects in representational similarity analysis: what (not) to do with the diagonal. Neuroimage 148, 197–200. 10.1016/j.neuroimage.2016.12.079 28069538

[B33] Saarimäki H. Gotsopoulos A. Jääskeläinen I. P. Lampinen J. Vuilleumier P. Hari R.. (2016). Discrete neural signatures of basic emotions. Cereb. Cortex 26, 2563–2573. 10.1093/cercor/bhv086 25924952

[B34] Sallet J. Mars R. B. Noonan M. P. Neubert F.-X. Jbabdi S. O’Reilly J. X.. (2013). The organization of dorsal frontal cortex in humans and macaques. J. Neurosci. 33, 12255–12274. 10.1523/JNEUROSCI.5108-12.2013 23884933 PMC3744647

[B35] Sammler D. Grosbras M.-H. Anwander A. Bestelmeyer P. E. G. Belin P. (2015). Dorsal and ventral pathways for prosody. Curr. Biol. 25, 3079–3085. 10.1016/j.cub.2015.10.009 26549262

[B36] Sauter D. A. Eisner F. Ekman P. Scott S. K. (2010). Cross-cultural recognition of basic emotions through nonverbal emotional vocalizations. Proc. Natl. Acad. Sci. U S A 107, 2408–2412. 10.1073/pnas.0908239106 20133790 PMC2823868

[B37] Saxe R. Moran J. M. Scholz J. Gabrieli J. (2006). Overlapping and non-overlapping brain regions for theory of mind and self reflection in individual subjects. Soc. Cogn. Affect. Neurosci. 1, 229–234. 10.1093/scan/nsl034 18985110 PMC2555418

[B38] Schurz M. Radua J. Aichhorn M. Richlan F. Perner J. (2014). Fractionating theory of mind: a meta-analysis of functional brain imaging studies. Neurosci. Biobehav. Rev. 42, 9–34. 10.1016/j.neubiorev.2014.01.009 24486722

[B39] Schwarzlose R. F. Baker C. I. Kanwisher N. (2005). Separate face and body selectivity on the fusiform gyrus. J. Neurosci. 25, 11055–11059. 10.1523/JNEUROSCI.2621-05.2005 16306418 PMC6725864

[B40] Sul S. Tobler P. N. Hein G. Leiberg S. Jung D. Fehr E.. (2015). Spatial gradient in value representation along the medial prefrontal cortex reflects individual differences in prosociality. Proc. Natl. Acad. Sci. U S A 112, 7851–7856. 10.1073/pnas.1423895112 26056280 PMC4485092

[B41] Vuilleumier P. Pourtois G. (2007). Distributed and interactive brain mechanisms during emotion face perception: evidence from functional neuroimaging. Neuropsychologia 45, 174–194. 10.1016/j.neuropsychologia.2006.06.003 16854439

[B42] Wager T. D. Kang J. Johnson T. D. Nichols T. E. Satpute A. B. Barrett L. F. (2015). A bayesian model of category-specific emotional brain responses. PLoS Comput. Biol. 11:e1004066. 10.1371/journal.pcbi.1004066 25853490 PMC4390279

